# Longitudinal pattern of bullying and cyberbullying victimization and perpetration in adolescence: a multi-method approach from the Bullying and Youth Mental Health Naples Study (BYMHNS)

**DOI:** 10.1186/s40359-026-04885-9

**Published:** 2026-07-03

**Authors:** Francesca Mottola, Carla Nasti, Matthew R. Broome, Antonio Pascotto, Katia Russo, Raffaella Iuliano, Margherita Siciliano, Simone Pisano, Vincenzo Paolo Senese, Gennaro Catone

**Affiliations:** 1 Department of Educational, Psychological and Communication Sciences, University “Suor Orsola Benincasa”, Naples, Italy; 2 Institute for Mental Health, School of Psychology, University of Birmingham, Birmingham, UK; 3 Department of Mental and Physical Health and Preventive Medicine, University of Campania “Luigi Vanvitelli”, Naples, Italy; 4 Ospedale del Mare” Hospital, Naples, Italy; 5 Department of Translational Medical Science, Federico II University, Naples, Italy; 6 Department of Psychology, University of Campania “Luigi Vanvitelli”, Caserta, Italy

**Keywords:** Bullying, Cyberbullying, Adolescence, Longitudinal Study, Assessment Methods

## Abstract

**Background:**

Bullying and cyberbullying are well recognized mental health risk factors and are of major public health concern during adolescence, yet inconsistencies in assessment methods and longitudinal design hinder accurate prevalence estimation and understanding of developmental trajectories. This longitudinal study aimed to: (a) compare prevalence estimates of bullying and cyberbullying using single-item versus multi-item assessments; (b) assess developmental changes in victimization and perpetration from 6th to 8th grade (ages 10–13).

**Methods:**

A cohort of 788 Italian middle school students (50.5% male) completed assessments in sixth (2015–2016) and eighth grade (2017–2018). Bullying and cyberbullying were measured with single-item and multi-item self-report instruments. Analyses included descriptive statistics, Pearson’s correlation alongside point-biserial correlations, McNemar’s tests, paired t-tests, generalized estimating equation models, mixed repeated-measures ANOVAs, Stuart–Maxwell tests, and intraclass correlation coefficients.

**Results:**

Single- and multi-item measures showed moderate convergence (*r* = .36–0.51). Single-item measures primarily capture global self-identification with victim or perpetrator roles, whereas multi-item measures provide greater specificity regarding the frequency, context, and modality of bullying. Measurement method influenced developmental trends: bullying victimization decreased as children got older using single-item tools (χ² = 24.59, *p* < .001) but remained stable with multi-item, while perpetration increased only via multi-item (d = -0.28). Cyberbullying findings were more tentative: single-item victimization decreased, multi-item victimization remained stable, and multi-item perpetration showed only a small increase. Supplementary analyses showed no significant Time × Sex interactions, and school-level clustering was negligible.

**Conclusions:**

Findings highlight that bullying assessment methodological choices strongly influence prevalence and developmental estimates. Multi-method longitudinal designs are crucial for valid assessment and effective prevention.

**Supplementary Information:**

The online version contains supplementary material available at https://doi.org/10.1186/s40359-026-04885-9.

## Background

Bullying involvement, whether as a victim, perpetrator, or both, is a major public health concern. It affects about one-third of school-aged adolescents worldwide [1–3]. Traditional bullying, defined as repeated aggressive behaviour involving an intention to cause physical, social, or emotional harm and an unequal distribution of power between the perpetrator and the victim [4, 5], has been extensively documented across physical, verbal, relational, and property-directed modalities. The rapid expansion of digital communication technologies has given rise to cyberbullying, a parallel yet distinct form of peer aggression perpetrated through electronic media [2, 6]. Cyberbullying shares core features with traditional bullying but adds unique elements that can amplify its impact. Indeed, perpetrators can conceal their identity, send messages at any time, and circulate content indefinitely across multiple platforms, potentially increasing victims’ feelings of shame. Moreover, cyberbullying can occur anywhere, making it difficult for victims to escape harassment [7–10]. Understanding how these two forms of victimization occur simultaneously, evolve across development, and relate to psychological adjustment remains a critical research priority. However, accurately estimating their prevalence and developmental trajectories, is substantially constrained by inconsistencies in assessment methods, particularly the choice between single-item and multi-item measures, a methodological gap that remains poorly understood in longitudinal research. Epidemiological studies indicate that bullying is widespread among school-aged populations. In a meta-analysis involving 42 studies, Li and colleagues [11] reported a mean victimization rate of 24.32% for traditional bullying and 11.10% for cyberbullying. Importantly, the two forms frequently co-occur, suggesting shared risk mechanisms [12–14].

These global patterns are reflected in national contexts, although with important regional and temporal variations. In Italy specifically, both national surveys and regional studies have documented substantial rates of bullying and cyberbullying, with notable temporal and regional variations. According to the 2023 report by the Italian National Institute of Statistics [15], approximately 21% of students aged 11–19 reported having experienced bullying in the past year. Consistent with these findings, the 2022 Health Behaviour in School-aged Children (HBSC) survey reported that fewer than 20% of adolescents aged 11–15 were identified as victims of bullying, with rates declining in older adolescents [16]. Beyond prevalence rates, research has also documented variation in the forms of bullying experienced [2, 17, 18]. Direct bullying, such as verbal insults and physical aggression, remains most common, while indirect forms, including social exclusion and defamation, affect a substantial proportion of students. Traditional assumptions have suggested that boys are more prone to be involved in direct, overt, and obtrusive forms of bullying, whereas girls typically exhibit bullying in indirect, covert, and subtle ways [19, 20]. Instead, a recent study [21] found that boys engage in both physical and relational bullying, whereas girls engage mainly in relational and less physically aggressive bullying. Moreover, regional analyses reveal marked differences, with northern regions reporting higher prevalence than southern regions across both bullying modalities [22]. In the Campania region, our previous cross-sectional investigations within the Bullying and Youth Mental Health Naples Study (BYMHNS) documented substantial prevalence rates among middle school students [23]. For traditional bullying, 37.1% reported victimization (25.7% once or twice, 11.4% three or more times) and 20.9% reported perpetration (15.7% once or twice, 5.1% three or more times) in the preceding six months [23]. For cyberbullying, 13.2% reported victimization and 7.8% reported perpetration during the school year [24]. While these baseline data established substantial prevalence rates in this southern Italian context, they left unresolved critical questions about developmental trajectories and measurement consistency over time. Longitudinal research shows that bullying peaks during early adolescence, particularly in middle school, after which rates of victimization typically decline while perpetration patterns display greater variability [17, 18, 25]. However, developmental trajectories of cyberbullying during this critical period remain less well characterized, particularly in Italian samples.

Despite this growing body of evidence, several epidemiological and methodological issues remain unresolved. One plausible explanation for the wide variability in reported prevalence is the lack of uniform definitions across studies and the challenges of multicultural comparisons, coupled with methodological limitations related to assessment procedures [1]. Many investigations have relied on small, convenience samples drawn from different regions and languages; however, large-scale representative samples including thousands of individuals are needed for more accurate population estimates. Comparability is further complicated by heterogeneity in measurement tools and study aims, indeed, some studies focused on prevalence estimation, others on evaluating prevention programs [26]. A central methodological debate concerns the optimal assessment strategy. A comprehensive review of bullying measures highlighted substantial methodological differences and recommended shared definition-based terminology, explicit timeframes, and the use of concrete behavioural examples in questionnaires [27]. Consistent with that review, roughly a quarter of studies have used a single dichotomous item, whereas the remaining three-quarters relied on multi-item scales, making cross-study comparisons difficult. A long-standing debate in the field concerns whether single-item questions, easy to administer but potentially less reliable, should give way to multi-item instruments, which generally show better validity and reliability [28]. Some studies [29] concluded that each approach has trade-offs: single-item measures are efficient and straightforward, imposing a lower burden on participants and often resulting in higher completion rates; whereas multi-item scales tend to reduce measurement error and provide richer coverage of the construct. Calls for closer psychometric comparisons among instruments remain only partially answered [30, 31], and the measurement properties of many commonly used tools have seldom been scrutinized. Despite these theoretical considerations, empirical comparisons remain limited and show substantial measurement discrepancies. Studies comparing single-item and multi-item approaches report agreement rates ranging from 44% to 77% [23, 32], with even larger discrepancies for cyberbullying [33, 34]. Systematic reviews continue to document wide variability in prevalence estimates across assessment methods, with rates varying by up to 40% points depending on measurement approach [35]. Even within the multi-item family, comparability issues persist: comparative validation work has shown that different multi-item cyberbullying scales (e.g., action-based vs. medium-based item sets) can measure the same latent construct, supporting comparability when psychometric equivalence is established—though many published studies still under-report reliability and validity evidence [36, 37].

In light of these considerations, despite extensive research on bullying and cyberbullying, critical gaps remain. First, although longitudinal studies have documented developmental trajectories in various contexts [2, 18, 38], most have relied on single assessment methods; multi-method longitudinal research is needed to determine whether different approaches yield convergent developmental patterns. Second, while cross-sectional studies have compared single-item and multi-item assessments [23, 32, 33], no longitudinal studies have integrated multi-method approaches for both traditional and cyberbullying within the same cohort. Third, to our knowledge, such investigations have not been conducted in Italy, where regional variations and limited prospective data underscore this need. Addressing these gaps is essential for: (a) establishing whether methodological choices influence prevalence estimates across time; (b) characterizing how bullying and cyberbullying involvement changes during the critical transition from early to mid-adolescence. Building on the abovementioned considerations and extending our previous cross-sectional findings [23] within the BYMHNS, the present study represents the longitudinal follow-up of that investigation while also examining additional aspects that were not addressed in the earlier work [23]. Its primary aim was to examine bullying and cyberbullying behaviors over time in the same school-based cohort of adolescents from the metropolitan area of Naples (Campania, Italy). Specifically, we aimed to: (a) investigate the prevalence of bullying and cyberbullying phenomena across time using both single- and multi-item measures and compare them; (b) estimate the developmental changes of bullying and cyberbullying victimization and perpetration from 6th to 8th grade using both single-and multi-item measures.

## Methods

### Study design

This longitudinal study represents the follow-up phase of the Bullying and Youth Mental Health Naples Study (BYMHNS), a school-based cohort investigation conducted within the Metropolitan area of Naples, in southern Italy. The baseline cross-sectional phase has been previously reported [23].

### Participants and procedure

**Fig. 1:**
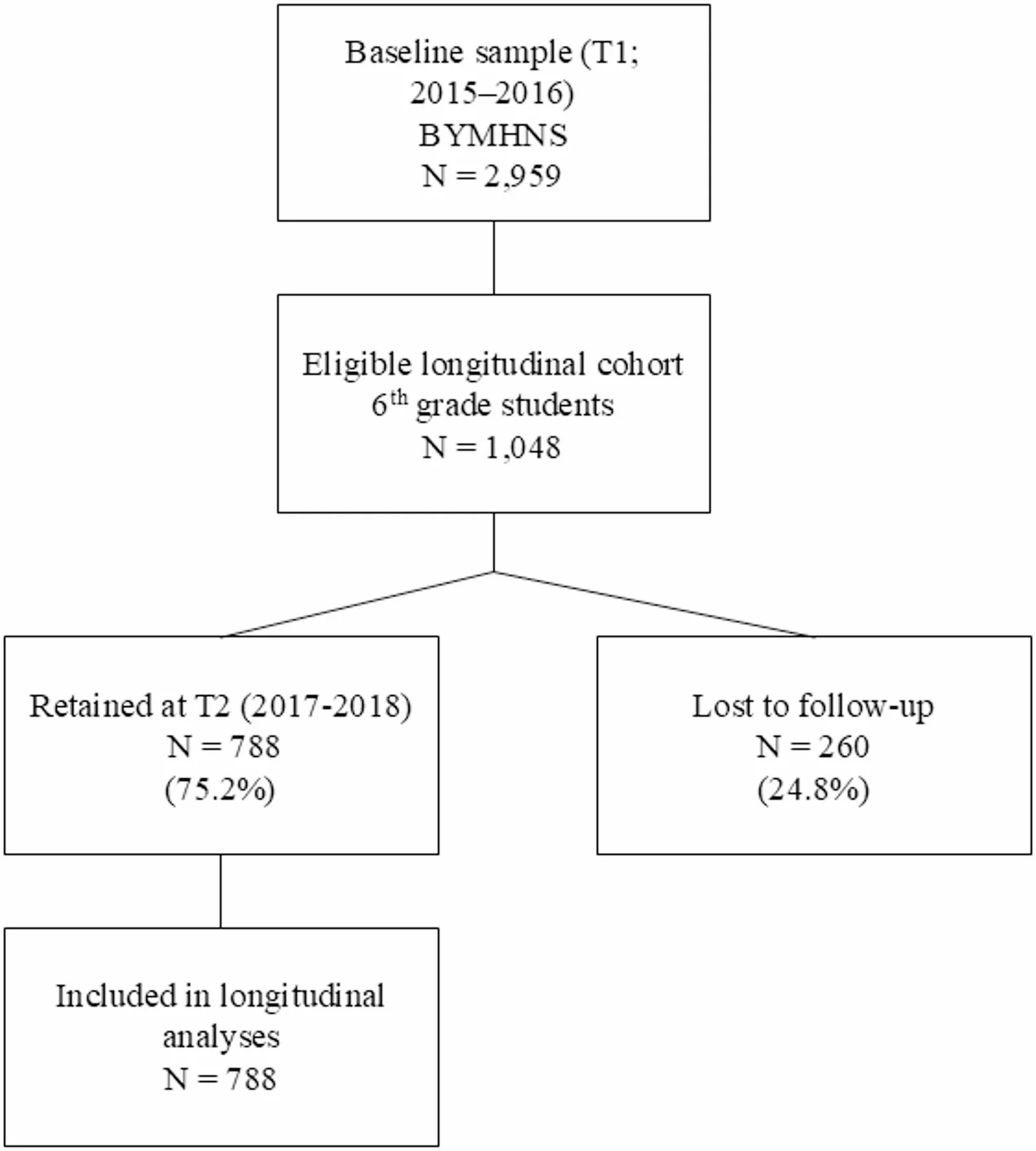
Flow diagram of participant recruitment and retention across study waves

The present study represents the longitudinal follow-up phase of the Bullying and Youth Mental Health Naples Study (BYMHNS), a large school-based investigation conducted in the metropolitan area of Naples, Southern Italy. The baseline assessment (T1; school year 2015–2016) included 2,959 middle-school students recruited from 11 schools in the Campania region, comprising students attending 6th grade (*n* = 1,048), 7th grade (*n* = 995), and 8th grade (*n* = 916) [23]. For the present longitudinal investigation, only students attending 6th grade at baseline were considered eligible for follow-up, as these participants could be reassessed during 8th grade two school years later (T2; school year 2017–2018). Thus, the eligible longitudinal cohort consisted of 1,048 students. At follow-up, 788 students (50.5% males) were successfully reassessed and matched across waves, yielding a retention rate of 75.2%. Attrition analyses based on the same BYMHNS longitudinal dataset have been previously reported [39]. In that study, retained and non-retained participants were compared on baseline demographic and psychopathological variables. Participants lost to follow-up showed higher externalizing symptoms at baseline, whereas no significant differences emerged for age, sex, internalizing symptoms, or mistrust. Because the previous attrition analysis did not specifically examine bullying and cyberbullying variables, and because missing data patterns may differ across measures, selective attrition related to peer aggression involvement cannot be entirely excluded. A total of 260 students (24.8%) were lost to follow-up because they were unavailable during data collection, had changed school, were absent on the day of assessment, did not provide consent, or could not be matched across time points where applicable. Participant flow across study waves is summarized in Fig. 1.

At T1, participants were attending 6th grade and their age distribution was as follows: 10–11 years (20.2%), 11–12 years (75.3%), and 12–13 years (4.6%). At T2, participants were attending 8th grade and ranged in age from 12 to 15 years.

### Recruitment procedure and informed consent

The study received ethical approval from the University of Campania “Luigi Vanvitelli” (approval number 500/29.04.2016). The study was conducted in accordance with the ethical principles of the Declaration of Helsinki. Following institutional agreements with eleven middle schools in the Campania region, written informed consent was obtained from all participants’ parents or legal guardians, and verbal assent was obtained from all students prior to data collection.

### Administration

Data collection took place during the school year 2015/2016 (T1–6th grade) and after two school years (2017/2018; T2–8th grade). Participants were administered in the paper self-report questionnaire in their classrooms by research assistants and in the presence of the teachers. In each school, a researcher supervised the questionnaire administration at all times to address any questions from participants.

### Measures

The data presented here were drawn from a section of the questionnaire assessing bullying and cyberbullying victimization and perpetration within the larger data collection (for more detailed information, see the Supplementary Material of Catone and colleagues’ study [23]). Bullying and cyberbullying phenomena were assessed using a multi-method approach incorporating both single- and multi-item questions. It should be noted that the single-item and multi-item measures differ not only in item number but also in recall period, response format, and construct specificity, and therefore do not represent directly equivalent operationalizations.

#### Demographic information

At the beginning of the questionnaire, information such as age, gender, class, section, and school were requested.

#### Bullying phenomenon measured with single-item question

Participants were asked whether they had ever been involved, either as victims or perpetrators, in bullying according to the definition provided by Olweus [4], which was presented by the researchers prior to the administration of questionnaire.

Furthermore, the definition was provided at the outset of the questionnaire using explanatory vignettes and examples, clarifying what constitutes bullying and what does not. The single-item question regarding victimization was “Have you ever been bullied?” with response options “No” (score = 0), or “Yes” (score = 1), instead the question regarding perpetration was “Have you ever bullied someone?” with the same response coding. Response to these two questions were used to derive lifetime bullying variables (victims/bullies). Moreover, respondents who answered “Yes” to both questions were asked additional questions to assess the frequency of these experiences over the preceding six months. Specifically, those reporting victimization were asked, “If you have been bullied how many times happened in the last six months?”, while those reporting perpetration were asked, “If you have bullied others how many times happened in the last six months?”. These items were used to assess current bullying victimization and perpetration. Response options included four categories: “Once”, “Twice”, “3–4 times” and “5–6 times or more”.

From the last two questions, three variables were derived to assess current bullying experiences for both being a victim and a perpetrator:

1. Absence: participants who answered “No” to the first question (score = 0);
2. Broad victimization or perpetration: participants who answered “Once” or “Twice” to the current bullying questions (score = 1);
3. Narrow victimization or perpetration: participants who answered “3–4 times” or “5–6 times or more” to the current bullying questions (score = 2).

These questions were derived from the “*Questionnaire on Bullying among Students at School*”, original version by Olweus [40], Whitney and Smith [41], translated and adapted in Italian language by Genta and colleagues [23, 42]. It should be noted that this gated structure, whereby only students confirming lifetime involvement were subsequently asked about current experiences. This distinction is critical for the interpretation of cross-method discrepancies and underscores the need for greater standardization in bullying assessment. Importantly, students who had experienced bullying recently but did not endorse lifetime involvement were not included in the current bullying measure.

#### Bullying victimization measured with multi-item questions

The Multidimensional Peer Victimization Scale (MPVS) [43] was translated into Italian by an expert and subsequently reviewed by an independent expert through a back-translation procedure [23]. The MPVS consists of 16 items divided in four subscales: (1) Physical victimization scale (e.g., “Have you ever been punched?”); (2) Verbal victimization scale (e.g., “Have you ever been called names (nicknames)?”); (3) Social manipulation scale (e.g., “Have you ever been made to argue with your friends?”); (4) Attacks on property scale (e.g., “Have you ever had something taken from you without your permission?”). Participants answered to each item using a three-point Likert scale that ranged from 0 to 2: “Not at all” (0), “Once” (1) and “More than once” (2). Each of the four subscales yields scores ranging from 0 to 8, and a total score is calculated as the sum of all item responses (range 0–32). Higher MPVS scores indicate higher victimization. All MPVS subscales and the total score showed good internal consistency at both T1 (*N* = 2959; 0.70 < α < 0.84; for more details, see the Supplementary Materials of Catone et al. [23] and T2 (*N* = 788; Physical Victimization: α = 0.75, Social Manipulation: α = 0.77, Verbal Victimization: α = 0.74, Attacks on Property: α = 0.69, and Total score: α = 0.83).

#### Bullying perpetration measured with multi-item questions

The Illinois Bully Scale (IBS) [44], an 18-item questionnaire, consists of three subscales: (1) Bully; (2) Victim; (3) Fighting. For this study, we focused only on the Bully subscale (item example: “In a group I teased other students”) as our primary interest was in bullying perpetration. Translation of the IBS Bully subscale into Italian was carried out by an expert and subsequently checked for accuracy by an independent expert through a back-translation procedure [23]. This subscale comprises nine items, each rated on a five-point response scale: “Never” (0), “1 or 2 times” (1), “3 or 4 times” (2), “5 or 6 times” (3) and “7 or more times” (4). Scores on this subscale have a possible range of 0–36. The Bully subscale showed a good internal consistency at both T1 (*N* = 2959; α = 0.82) and T2 (α = 0.82).

#### Cyberbullying phenomenon measured with single-item question

Before completing the self-report assessment, a definition of cyberbullying was provided to participants by the researchers and were then asked whether they had ever experienced or engaged in cyberbullying. The single-item measure of victimization was: “Have you experienced cyberbullying during this school year?” (possible answers were “No” - score = 0, “Yes, by friends outside the school” - score = 1, “Yes, by schoolmates” – score = 2, or “Yes, both by schoolmates and friends outside the school” – score = 3), while the question regarding perpetration was “Have you perpetrated cyberbullying during this school year?” (response options: “No” - score = 0; “Sometimes” – score = 1; or “Often” – score = 2).

#### Cyberbullying phenomenon measured with multi-item questions

The Smith Cyberbullying Self-Report Questionnaire [45], adapted for the Italian context by Bacchini [46], was used to assess cyberbullying. The questionnaire evaluates both victimization and perpetration experiences across eight different types of cyberbullying: (1) text messaging, (2) pictures, photos, or video clips, (3) phone calls, (4) email, (5) chat rooms, (6) instant messaging, (7) websites, and (8) other online means. Participants were asked to indicate how frequently each type of episode had occurred since the beginning of the school year. Answers were evaluated using a five-point Likert scale that ranged from “Never” (0) to “Several times a week” (4), with intermediate options of “1 or 2 times” (1), “2–3 times at month” (2), and “Once a week” (3). The scale showed adequate internal consistency at T1 (*N* = 2959; Victimization: α = 0.76; Perpetration: α = 0.81). At T2, internal consistency was lower, particularly for cyberbullying perpetration (*N* = 788; Victimization: α = 0.60; Perpetration: α = 0.50).

### Data analysis

First, for bullying and cyberbullying prevalence, results of both single- and multi-item approaches were analyzed. As regards the single-item approach, frequency distributions and percentage were considered. In particular, for bullying victimization and perpetration, frequencies distribution and percentage were computed for lifetime and current bullying. As regards multi-item approach, descriptive statistics were reported as means and standard deviations. Preliminarily, for single-item cyberbullying victimization and perpetration, answers were recoded in dummy variables (*Victimization*: “No” - score 0 remained unchanged; “Yes, by friends outside the school” - score 1 remained unchanged; “Yes, by schoolmates” – score 2 was recoded in score 1; “Yes, both by schoolmates and friends outside the school” – score 3 was recoded in score 1. *Perpetration*: “No” - score 0 remained unchanged; “Sometimes” - score 1 remained unchanged; “Often” – score 2 was recoded in score 1). Then, to assess the agreement between the single- and the multi-item approaches for both bullying and cyberbullying phenomena, the point-biserial correlations were computed. Effect sizes were estimated using Cohen’s d, and Pearson correlations were used to assess the associations between scores at Time 1 and Time 2 aimed at evaluating temporal stability. Third, concurrent associations between traditional bullying and cyberbullying experiences, as both victims and perpetrators, were examined separately at Time 1 and Time 2 using Pearson correlation coefficients for both single-item (interpreted as phi (φ) coefficients) and multi-item measures. These analyses were conducted to describe the concurrent overlap between offline and online forms of peer aggression at each assessment point. Correlation coefficients were interpreted descriptively. Because the present study was not designed to formally compare dependent overlapping correlations, no inferential tests of differences between correlation magnitudes (e.g., Steiger’s z or Hotelling-Williams tests) were conducted. Fourth, to verify whether and eventually how bullying/cyberbullying victimization and perpetration changed from 6th to 8th grade within the same adolescents, results of both single- and multi-item approaches were analyzed. As regards the single-item approach, McNemar’s tests were computed. For prevalence-oriented analyses and to ensure comparability with dichotomous bullying indicators, the single-item cyberbullying variables were recoded into binary indicators reflecting the presence versus absence of cyberbullying involvement. This choice was also informed by the low endorsement frequency of some response categories. However, we acknowledge that dichotomization necessarily entails loss of information, reduced variability, and may reduce sensitivity to meaningful differences in response intensity, consistent with longstanding methodological concerns regarding the dichotomization of variables [47, 48]. To address this limitation, supplementary analyses retaining the original ordinal response structure were conducted using Stuart–Maxwell tests, which are appropriate for paired multinomial categorical data (see Supplementary Tables S5–S6). Importantly, conclusions derived from the ordinal analyses were broadly consistent with those obtained using the dichotomized indicators. As regards the multi-items approach, paired sample t-tests were performed. To address potential sex differences in developmental patterns, supplementary sex-stratified analyses were conducted. For dichotomous single-item indicators, generalized estimating equation (GEE) logistic models were used to test Time × Sex interactions. For continuous multi-item outcomes, mixed repeated-measures ANOVAs were conducted with Time as the within-subject factor and Sex as the between-subject factor. These analyses were conducted for the principal bullying and cyberbullying victimization and perpetration outcomes and are reported in Supplementary Tables S7 and S8. To assess potential school-level clustering, intraclass correlation coefficients (ICCs) were estimated for the principal continuous multi-item outcomes at T1 and T2 using unconditional random-intercept models with students nested within schools. ICCs were estimated only for multi-item continuous measures, for which conventional ICC estimation is most appropriate. Results are reported in Supplementary Table S9. Finally, to control for familywise Type I error, the False Discovery Rate (FDR) procedure was applied to adjust p-values [49]. All statistical analyses were performed using IBM SPSS Statistics version 26.0 and R version 4.3.3.

## Results

### Lifetime bullying categories (single-item assessment)

In line with the definition of lifetime bullying, at T1, 359 (45.6%) students reported having experienced one episode of victimization during their life, while 159 (20.2%) students reported at least one lifetime episode of bullying perpetration.

At T2, 269 (34.1%) students reported having experienced at least one episode of victimization in their life, while 156 (19.8%) students reported having engaged at least in one episode of bullying in their life. The observed reduction in lifetime victimization prevalence from T1 to T2 within the same cohort warrants interpretive caution. Given that lifetime measures assess cumulative experience, a lower prevalence at follow-up cannot reflect an actual reduction in past exposure. This pattern is most likely attributable to measurement instability, including shifts in self-definitional thresholds, changes in the subjective interpretation of the bullying label, or differential recall across time points.

### Current bullying categories (single-item assessment)

In line with the definition of current bullying, at T1, in the last six months, 330 (41.9%) students reported experiencing victimization, of which 231 students once or twice (29.3%, broad category), and 99 students three or more times (12.6%, narrow category). 148 (18.9%) students reported perpetrating bullying in the last six months, of whom 120 students did so once or twice (15.2%, broad category), and 28 students three or more times (3.6%, narrow category). Comparison between lifetime and current involvement showed a difference of 29 students (3.7%) for victimization, namely, participants who reported lifetime victimization but not in the past six months. Similarly, a difference of 11 students (1.3%) was observed for perpetration, representing those who reported lifetime perpetration but no episodes in the last six months.

At T2, 192 (24.4%) students reported experiencing victimization in the previous six months. Among them, 121 students indicated being victimized once or twice (15.4%, broad category), whereas 71 students reported three or more episodes (9%, narrow category). 131 (16.6%) students reported involvement in bullying perpetration during the previous six months, of whom 90 students did so once or twice (11.4%, broad category) and 41 students three or more times (5.2%, narrow category). Comparison between lifetime and current involvement revealed a difference of 77 students (9.7%) for victimization, that is those who reported lifetime victimization but not in the past six months, and a difference of 25 students (3.2%) for perpetration, representing participants involved in lifetime bullying perpetration but not in the previous six months.

### Bullying phenomenon (multi-item assessment) and association between single- and multi-item assessments

Table 1 shows descriptive statistics (i.e., means and standard deviations) for MPVS and IBS-B across broad and narrow categories of bullying victimization and perpetration at both time points. As reported in the Additional file (Table S1), the single-item measures showed moderate correlations with the corresponding multi-item scales at both Time 1 and Time 2.

**Table 1:** Means and standard deviations for MPVS and IBS-B (Time 1 and Time 2)

Time 1	Broad victims (*N* = 231)	Narrow victims (*N* = 99)
MPVS, *mean (SD)*	10.90 (5.76)	15.82 (6.33)
Physical victimization, *mean (SD)*	1.38 (1.83)	2.30 (2.62)
Verbal victimization, *mean (SD)*	3.92 (2.24)	5.69 (2.13)
Social manipulation, *mean (SD)*	2.98 (2.33)	4.26 (2.41)
Attacks on property, *mean (SD)*	2.62 (2.18)	3.57 (2.41)
	Broad perpetrators (*N* = 120)	Narrow perpetrators (*N* = 28)
IBS-B, *mean (SD)*	4.23 (3.06)	11.11 (7.79)
Time 2	Broad victims (*N* = 121)	Narrow victims (*N* = 71)
MPVS, *mean (SD)*	11.71 (5.62)	14.99 (6.24)
Physical victimization, *mean (SD)*	1.30 (1.77)	2.04 (2.33)
Verbal victimization, *mean (SD)*	4.51 (2.27)	5.97 (1.76)
Social manipulation, *mean (SD)*	2.97 (2.41)	3.58 (2.74)
Attacks on property, *mean (SD)*	2.94 (2.03)	3.39 (2.48)
	Broad perpetrators (*N* = 90)	Narrow perpetrators (*N* = 41)
IBS-B, *mean (SD)*	5.61 (4.42)	11.88 (7.80)

### Concurrent associations between bullying and cyberbullying (Single-Item and Multi-Item) at both time 1 and time 2

In addition to descriptive analyses, concurrent associations between traditional bullying and cyberbullying were examined at both T1 and T2 using single-item and multi-item measures (see Supplementary Tables S3 and S4).

Single-item correlations were moderate, with bullying victimization related to cyberbullying victimization (*r* ≈ .28) and bullying perpetration to cyberbullying perpetration (*r* ≈ .32). Multi-item correlations appeared descriptively larger (victimization *r* = .45–0.49; perpetration *r* = .60–0.63). Overall, associations appeared numerically higher for perpetration than for victimization, suggesting a stable overlap between offline and online aggression.

### Longitudinal Patterns of Bullying Victimization and Perpetration from 6th to 8th grade

Table 2 presents the outcomes of McNemar’s test comparing self-reported bullying victimization and perpetration using single-item assessments across two time points.

**Table 2:** McNemar’s test results for bullying victimization and perpetration (single-item) at two time-points

Bullying				
*Victimization*			McNemar’s test	
Have you ever been bullied?	No	Yes	*N* (total)	776
No	321	101	*X* ^*2*^	24.59
Yes	186	168	Asymp. Sig.	< 0.001
*Perpetration*				
Have you ever made bullying?	No	Yes	*N* (total)	779
No	536	86	*X* ^*2*^	0.000
Yes	87	70	Asymp. Sig.	1.000

*p*-value corrected for FDR

McNemar’s test showed a statistically significant difference in self-reported bullying victimization from 6th to 8th grade (χ² = 24.59, *p* < .001). Among 776 students, 321 consistently reported no victimization, 168 consistently reported victimization, 101 moved from no report at T1 to reporting victimization at T2, and 186 had experienced victimization at T1 but not at T2.

For bullying perpetration, McNemar’s test revealed no detectable change in proportion of students reporting perpetration across the two time points (χ² = 0.000, *p* = 1.000). Among 779 students, 536 consistently reported no perpetration, 70 consistently reported perpetration, 86 moved from no reported perpetration at T1 to reporting perpetration at T2, and 87 reported perpetrations at T1 but not at T2.

Paired sample t-tests (see Table 3) examined changes in continuous multi-item scores across the two time-points. Given that single- and multi-item approaches operate on different scales, binary prevalence versus continuous mean scores, these results should be interpreted as descriptively distinct patterns rather than directly comparable developmental trajectories. Paired sample t-tests showed modest but statistically significant reductions in physical victimization (*t*(786) = 3.60, *p* < .001, *d* = 0.13) and social manipulation (*t*(786) = 2.82, *p* < .01, *d* = 0.10). Verbal victimization and attacks on property did not change significantly, and the overall victimization composite score demonstrated a non-significant reduction (*t*(786) = 1.91, *p* > .05, *d* = 0.07). Correlations between the two time points ranged from *r* = .33 to *r* = .46 (FDR corrected for *p* < .001), indicating moderate temporal stability across victimization dimensions. In contrast, bullying perpetration showed a significant increase from T1 to T2 (*t*(786) = -7.85, *p* < .001, *d* = -0.28). The correlation between the two time-points scores was moderate and significant (*r* = .40, *p* < .001), indicating stability in individual differences across assessments.

**Table 3:** Paired sample t-tests for bullying victimization and perpetration (multi-item) across two time points

Bullying Victimization (*N* = 787)						
	Time 1 Mean (SD)	Time 2 Mean (SD)	t-value	df	Cohen’s d	*r*-value
Physical Victimization	1.05 (1.73)	0.81 (1.51)	3.60***	786	0.13	0.33***
Social Manipulation	2.38 (2.31)	2.13 (2.24)	2.82**	786	0.10	0.38***
Verbal Victimization	3.14 (2.49)	3.17 (2.53)	− 0.27	786	− 0.01	0.43***
Attacks on Property	2.19 (2.07)	2.23 (2.03)	− 0.45	786	− 0.02	0.33***
Total score	8.76 (6.32)	8.33 (6.06)	1.91	786	0.07	0.46***
Bullying Perpetration (*N* = 787)						
	Time 1 Mean (SD)	Time 2 Mean (SD)	t-value	df	Cohen’s d	r-value
IBS-B	2.35 (3.35)	3.59 (4.55)	-7.85***	786	− 0.28	0.40***

***FDR corrected *p*<.001; **FDR corrected *p*<.01

### Cyberbullying phenomenon (single-item assessment)

#### Victimization

In accordance with the definition of cyberbullying, at T1, 684 (86.8%) students reported they did not suffer from an episode of cyberbullying during the ongoing academic year, instead, 104 students (13.2%) reported they suffered from an episode of cyberbullying, of which 42 students (5.3%) by friends outside the school, 47 students (6%) by schoolmates and 15 students (1.9%) both by schoolmates and friends outside school.

At T2, 728 (92.4%) students reported they did not suffer from an episode of cyberbullying during the ongoing academic year, instead, 53 students (6.6%) reported they suffered from an episode of cyberbullying, of which 24 students (3%) by friends outside the school, 20 (2.5%) by schoolmates, 9 students (1.1%) both by schoolmates and friends outside school. Seven participants (0.7%) did not answer.

#### Perpetration

At T1, 732 (92.9%) students reported they did not perpetrate an episode of cyberbullying during the ongoing academic year, instead, 50 students (6.3%) reported they perpetrated an episode of cyberbullying sometimes and 6 students (0.8%) reported they often perpetrated an episode of cyberbullying.

At T2, 733 (93%) students reported they did not perpetrate an episode of cyberbullying during the ongoing academic year, instead, 36 students (4.6%) reported they perpetrated an episode of cyberbullying sometimes and 2 students (0.3%) reported they often perpetrated an episode of cyberbullying. Seventeen participants (2.2%) did not answer.

### Cyberbullying phenomenon (multi-item assessment) and relation between single and multi-item assessments

Table 4 shows descriptive statistics (i.e., means and standard deviations) for cyberbullying victimization and perpetration across broad and narrow categories at both time points. As reported in the Additional file (Table S2), single-item cyberbullying measures showed moderate correlations with the corresponding multi-item scales at both Time 1 and Time 2.

**Table 4:** Means and standard deviations for cyberbullying victimization and perpetration (Time 1 and Time 2)

Scale	Time 1	Time 2
	Mean (SD)	Mean (SD)
Cyberbullying victimization^a^	1.58 (2.79)	1.54 (2.05)
Cyberbullying perpetration^b^	0.70 (1.77)	0.93 (1.46)

^a^Sample size: T1 = 788 and T2 = 784; ^b^Sample size: T1 = 788 and T2 = 785

### Longitudinal patterns of cyberbullying victimization and perpetration from 6th to 8th grade

Regarding the single-item measure of cyberbullying victimization (see Table 5), McNemar’s test indicated a significant change between grade 6 and grade 8, *χ²*(1) = 20.04, *p* < .001. Among 781 students, 646 consistently reported no victimization, 20 consistently reported victimization, 33 moved from no report at T1 to reporting victimization at T2, and 82 had experienced victimization at T1 but not at T2. Supplementary ordinal analyses retaining the original response categories yielded substantively consistent findings. Specifically, Stuart–Maxwell tests indicated a significant change in the distribution of cyberbullying victimization responses across time, χ²(3) = 21.26, *p* < .001 (see Supplementary Table S5).

**Table 5:** McNemar’s test results for cyberbullying victimization and perpetration (single-item) at two time points

Cyberbullying				
*Victimization*			McNemar’s test	
Have you experienced cyberbullying during this school year?	No	Yes	*N* (total)	781
No	646	33	*X* ^*2*^	20.04
Yes	82	20	Asymp. Sig.	< 0.001
*Perpetration*				
Have you perpetrated cyberbullying during this school year?	No	Yes	*N* (total)	771
No	690	26	*X* ^*2*^	3.71
Yes	43	12	Asymp. Sig.	0.054

*p*-value corrected for FDR

For cyberbullying perpetration, McNemar’s test did not reveal a statistically significant change, *χ²*(1) = 3.71, *p* = .054. Among 771 students, 690 consistently reported no perpetration, 12 consistently reported perpetration, 26 moved from no reported perpetration at T1 to reporting perpetration at T2, and 43 reported perpetration at T1 but not at T2. Likewise, ordinal analyses of cyberbullying perpetration responses yielded conclusions broadly consistent with the dichotomized analyses. Stuart–Maxwell tests did not indicate a statistically significant change in the distribution of perpetration responses over time, χ²(2) = 5.35, *p* = .069 (see Supplementary Table S6).

Turning to the multi-item approach, mean scores provided a more nuanced picture (see Table 6). For cyberbullying victimization, the mean levels remained stable from T1 (M = 1.58, SD = 2.80) to T2 (M = 1.54, SD = 2.05), with the paired sample t-test revealing no statistically significant difference, *t*(783) = 0.35, *p* > .05.

**Table 6:** Paired sample t-tests for cyberbullying victimization and perpetration (multi-item) across two time points

Cyberbullying						
	Time 1 Mean (SD)	Time 2 Mean (SD)	t-value	Df	Cohen’s d	*r*-value
Victimization (*N* = 784)	1.58 (2.80)	1.54 (2.05)	0.35	783	0.01	0.32***
Perpetration (*N* = 785)	0.70 (1.76)	0.93 (1.46)	-3.39**	784	− 0.12	0.31***

***FDR corrected *p*<.001; **FDR corrected *p*<.01

In contrast, cyberbullying perpetration showed a statistically significant but small increase over time, with mean scores rising from T1 (M = 0.70, SD = 1.76) to T2 (M = 0.93, SD = 1.46), *t*(784) = -3.39, *p* < .01, *d* = − 0.12.

### Supplementary sex-specific and clustering analyses

Supplementary analyses examined whether longitudinal patterns differed by sex. For dichotomous single-item indicators, GEE logistic models showed no significant Time × Sex interactions for bullying victimization, bullying perpetration, cyberbullying victimization, or cyberbullying perpetration after FDR correction (see Supplementary Table S7). Significant main effects of Time were retained for bullying victimization and cyberbullying victimization, whereas the Time effect for cyberbullying perpetration did not remain significant after FDR correction. A significant main effect of Sex was retained for bullying perpetration and cyberbullying victimization. For multi-item outcomes, mixed repeated-measures ANOVAs similarly showed no significant Time × Sex interactions across the four principal outcomes after FDR correction (see Supplementary Table S8). Overall, the absence of significant Time × Sex interactions suggests that longitudinal patterns were broadly comparable across boys and girls.

To evaluate school-level clustering, ICCs were estimated for the principal continuous multi-item outcomes. ICCs were negligible across all outcomes, ranging from 0.000 to 0.036 (see Supplementary Table S9), suggesting minimal school-level dependence. 

## Discussion

This longitudinal investigation examined prevalence patterns, developmental trajectories, and prospective associations of bullying and cyberbullying among middle school students across a two-year period from 6th to 8th grade (10–14 years approximately). Employing parallel single-item and multi-item assessment methodologies, this research addressed two interrelated objectives: (a) investigate the prevalence of bullying and cyberbullying phenomena across time using both single- and multi-item approaches, and compare them; (b) estimate the developmental changes of bullying and cyberbullying victimization and perpetration from 6th to 8th grade using both single-item and multi-item approaches. This integrated methodological and developmental framework advances understanding of peer aggression dynamics during early adolescence.

Our findings revealed descriptively distinct patterns in both prevalence estimates and developmental trends as a function of assessment methodology and aggression modality. Because single- and multi-item approaches operate on fundamentally different scales, binary prevalence tested via McNemar’s test versus continuous mean scores tested via paired t-tests, direct statistical comparison of developmental trajectories across methods was not possible, and observed differences should be interpreted with appropriate caution. Importantly, the present comparison does not isolate the effect of item number per se, given that the single-item and multi-item measures differ along multiple dimensions, including recall period, response format, and construct specificity. Accordingly, the observed differences should be interpreted as reflecting distinct operationalizations of bullying and cyberbullying rather than a pure format effect. Consistent with prior meta-analytic evidence [1], victimization prevalence consistently exceeded perpetration across all measurement approaches. Single-item lifetime measures showed an apparent reduction in victimization prevalence from sixth to eighth grade; however, this pattern should not be interpreted as evidence of developmental decline, as a cumulative lifetime measure cannot logically decrease within the same cohort. Rather than reflecting genuine behavioral change, this pattern likely indicates instability in self-report processes, highlighting the limitations of global self-identification measures for capturing developmental trajectories. By contrast, multi-item assessments, which measure behavioural frequency rather than global role identification, may provide a more informative basis for developmental inference, although their psychometric properties should be carefully considered: perpetration showed a modest increase over time, consistent with longitudinal studies documenting the persistence or escalation of indirect and relational forms of aggression during early adolescence [17, 49–51]. Multi-item assessments revealed that physical victimization and social manipulation decreased, while verbal victimization and attacks on property persisted. Developmental theories suggest that as adolescents acquire more advanced emotional regulation and social-cognitive skills, overt aggression diminishes while covert forms persist [52]. This trajectory is consistent with Björkqvist et al.‘s developmental model of aggression [53], which posits that as verbal and cognitive capacities mature, physical aggression gives way to increasingly indirect and relational strategies. Similarly, Crick and Dodge’s *Social Information Processing Model* [54] suggests that with age, adolescents become more proficient at encoding social cues and selecting relationally sophisticated aggressive responses, rendering covert forms of aggression both more accessible and less likely to invite adult sanctions. This persistence may reflect the stability of social hierarchies and peer reputation [55, 56], where verbal and relational forms remain socially functional or less likely to be sanctioned. These unmeasured influences likely contribute to the variability observed in prevalence estimates and developmental trends across aggression forms and assessment methods.

Supplementary analyses further indicated that these longitudinal patterns did not differ significantly by sex. Although sex differences in mean levels were observed for some perpetration-related indicators, particularly bullying perpetration, Time × Sex interactions were not significant across either single-item or multi-item outcomes. Thus, the observed developmental patterns appeared broadly comparable for boys and girls.

Turning to cyberbullying, developmental patterns diverged from those observed for traditional bullying. Descriptively, single-item self-reported victimization showed a reduction, whereas multi-item perpetration scores increased and multi-item victimization remained stable. These descriptively divergent patterns across methods suggest that behavioural frequency inventories and global self-perception items may capture different aspects of cyberbullying involvement; however, given the non-equivalence of the analytical frameworks employed, these differences cannot be formally attributed to measurement sensitivity alone. Importantly, supplementary ordinal analyses retaining the original cyberbullying response categories yielded findings broadly consistent with those obtained using dichotomized indicators, supporting the general consistency of the main conclusions despite the unavoidable loss of information associated with dichotomization. However, the observed increase in multi-item cyberbullying perpetration should be interpreted with considerable caution. The internal consistency of the perpetration scale decreased substantially from T1 (α = 0.81) to T2 (α = 0.50), raising concerns regarding measurement stability across waves. Consequently, it cannot be excluded that part of the observed longitudinal change reflects psychometric instability or measurement error rather than genuine behavioral variation. A similar, although less pronounced, concern applies to cyberbullying victimization, whose internal consistency at T2 was comparatively lower (α = 0.60), suggesting that longitudinal comparisons should be interpreted cautiously. Moderate convergence between single- and multi-item measures was suggestive of convergent validity, while also suggesting that each methodological approach may capture partially distinct facets of bullying involvement. In addition, concurrent associations between traditional bullying and cyberbullying were generally positive at both T1 and T2, with correlations appearing descriptively larger for perpetration than for victimization. This indicates a stable overlap between offline and online forms of aggression, suggesting that students involved in one domain are at higher risk of involvement in the other. However, no formal statistical comparison between dependent correlations was conducted; therefore, differences in correlation magnitudes should be interpreted descriptively.

These findings corroborate theoretical distinctions wherein single-item measures predominantly capture global self-identification with victim or perpetrator roles, whereas multi-item behavioral inventories afford greater specificity regarding frequency, context, and modality of bullying [57]. Importantly, the observed divergences in prevalence and developmental estimates should not be attributed solely to the number of items. The instruments compared in this study differ across multiple dimensions, including recall period, response format, scoring threshold, and construct operationalization, and therefore reflect broader measurement non-equivalence rather than an isolated item-format effect. This distinction is critical for the interpretation of cross-method discrepancies and underscores the need for greater standardization in bullying assessment. A further source of non-equivalence concerns the gated structure of the single-item current bullying measure, which was administered only to students who had previously endorsed lifetime involvement. This design likely underestimates current victimization and perpetration rates and further constrains direct comparison with multi-item instruments, which were administered to all participants without prior screening. Consequently, parallel deployment of both assessment strategies yields complementary information: single-items efficiently identify individuals recognizing their role-involvement, while multi-item scales provide granular behavioral profiles essential for intervention targeting. It should also be noted that self-report measures of both types are susceptible to social desirability bias and recall inaccuracies, which may differentially affect single- and multi-item formats. Single-item global questions may be more prone to underreporting due to stigma associated with role identification, whereas multi-item behavioral inventories, by querying specific acts rather than labels, may partially mitigate this bias [27, 28]. This convergent-divergent validity pattern highlights the potential value of multi-method assessment frameworks in bullying research and raises caution regarding prevalence studies relying exclusively on a single method approach.

Despite methodological strengths, including a large sample, longitudinal design, and multi-method assessment, some limitations must be acknowledged. First, exclusive reliance on self-report assessments may introduce recall bias and confounding effects related to social desirability, though self-report remains standard and often most valid for capturing subjective victimization experiences and covert perpetration behaviors. Second, the two-year assessment interval, while capturing critical early-to-mid-adolescent transitions, precludes examination of longer-term developmental cascades and limits causal inference regarding temporal precedence. Third, the study did not systematically assess cross-context co-occurrence patterns; given robust evidence of traditional-cyber bullying overlap [58], future investigations should apply person-centered methods, such as latent class analysis, to identify involvement typologies and cross-contextual risk pathways. Fourth, critical ecological moderators including family functioning, teacher support quality, peer network characteristics, and school climate indicators were not assessed, limiting understanding of contextual factors that may amplify or buffer individual vulnerability [59]. Fifth, potential selective attrition should be considered. Although attrition analyses previously conducted on the same BYMHNS longitudinal dataset suggested limited attrition effects, with retained and non-retained participants differing only in baseline externalizing symptoms and not in age, sex, internalizing symptoms, or mistrust [39], bullying and cyberbullying variables were not specifically examined in those analyses. Moreover, the present analytic sample was partially defined by the availability of bullying and cyberbullying data across waves, and missingness patterns may differ across measures. Therefore, selective attrition related to peer aggression involvement cannot be entirely excluded, and survivorship bias may have modestly influenced longitudinal estimates. Sixth, although participants were nested within schools, the present analyses did not employ multilevel longitudinal models. However, supplementary analyses indicated negligible school-level clustering across the principal multi-item outcomes (ICCs ranging from 0.000 to 0.036), suggesting that school-level dependence was unlikely to substantially influence the findings. Furthermore, clustering was evaluated at the school level only; class-level nesting could not be examined due to unavailable classroom identifiers across waves. Nevertheless, the relatively small number of participating schools may have limited the precision of between-school variance estimates, and future research involving larger school samples and classroom-level information should further investigate bullying trajectories using fully multilevel longitudinal approaches. Seventh, the internal consistency of the cyberbullying measures at T2 was relatively low, particularly for perpetration (α = 0.50) and, to a lesser extent, for victimization (α = 0.60). This limits confidence in longitudinal comparisons and raises the possibility of measurement instability across assessment waves. Moreover, although supplementary ordinal analyses yielded substantively consistent findings, the dichotomization of cyberbullying indicators may have reduced sensitivity to variation in response intensity. Accordingly, both the stability observed for cyberbullying victimization and the small increase observed for cyberbullying perpetration should be interpreted cautiously. Future studies should explicitly examine longitudinal measurement invariance to determine whether cyberbullying constructs are assessed equivalently across developmental stages.

To address these limitations and extend current findings, future research should prioritize four complementary directions. First, multi-informant, multi-method designs integrating self-report with peer nominations, teacher observations, and objective behavioral indices (including digital trace data for cyberbullying) would mitigate mono-method bias and enhance construct validity. Second, extended longitudinal follow-up through high school and emerging adulthood would elucidate long-term developmental trajectories, including persistence, escalation, desistance patterns, and distal psychosocial outcomes. Third, person-centered analytic frameworks (latent class/transition analysis, growth mixture modeling) could identify distinct developmental subgroups characterized by differential risk profiles and intervention responsiveness [44, 60].

## Conclusions

In conclusion, the present study contributes to a more nuanced understanding of bullying during early adolescence by highlighting how methodological choices and developmental factors intersect to shape bullying trajectories. The findings demonstrate that while certain forms of traditional victimization decline over time, others, especially verbal and relational forms, persist, and that cyberbullying perpetration showed a small and tentative increase in multi-item scores, although this finding should be interpreted cautiously given the low internal consistency of the measure at T2. By combining single-item and multi-item approaches, this research underscores the importance of multi-method assessment for capturing the multifaceted nature of bullying/cyberbullying.

The present findings have several practical implications for schools, mental-health professionals, and policymakers engaged in prevention of bullying. First, the study highlights the critical role of measurement in shaping conclusions about both the prevalence and developmental course of bullying and cyberbullying. Because single-item indicators tended to underestimate prevalence and produced developmental trends that diverged from those obtained through multi-item scales, school-based monitoring systems should prioritize validated multi-item measures. The use of consistent instruments across years is also essential, as variations in measurement tools may be mistaken for substantive behavioral change. Moreover, the provision of clear, standardized definitions prior to assessment appears necessary to minimize interpretive variability among students. Concretely, schools should implement systematic screening using validated multi-item instruments at least twice during the middle-school years, ideally at the transition into 6th grade and again at the end of 8th grade, to capture both the onset and developmental course of bullying involvement. Screening results should inform a tiered intervention approach, including universal prevention programs delivered to all students, selective interventions targeting those showing early signs of involvement, and indicated interventions for students already identified as victims or perpetrators. Given the observed increase in perpetration across the middle-school years, preventive efforts should be prioritized in early adolescence, before aggressive peer dynamics become consolidated. Second, the results have implications for the timing and focus of prevention. Although the single-item measure suggested a decline in victimization, the absence of comparable trends in the multi-item scales indicates that victimization remains a relevant concern across the middle-school years. Schools should therefore avoid reducing preventive efforts after early adolescence. In contrast, the multi-item scales indicated an increase in perpetration across the same period. However, the finding for cyberbullying perpetration was small and should be interpreted cautiously due to the low internal consistency of the measure at T2. Taken together, these findings suggest that interventions targeting aggression, emotion regulation, and peer-group dynamics may be particularly important during the transition from early to mid-adolescence. Cyberbullying prevention should specifically address digital literacy, online peer pressure, and awareness of the potential social and legal consequences of online aggression. Furthermore, the observed positive associations between offline and online forms of bullying highlight the importance of integrated prevention programs that address both offline and online aggression simultaneously. Third, results highlight the need of comprehensive training for teachers and school personnel, as well as for greater parental support and guidance. In particular, educators and caregivers should be equipped to understand and monitor children’s engagement with social media and digital communication platforms, given their relevance for the identification and prevention of cyberbullying. Given the modest convergence between measurement approaches, educators should be equipped to interpret assessment results cautiously and to integrate information from multiple sources, including student self-reports, teacher observations, and, when appropriate, parent perspectives. Such an approach can enhance the accuracy of identification and support decisions regarding intervention. Finally, the study raises considerations for national and institutional policies. Guidelines for bullying surveillance should incorporate multi-item, psychometrically robust instruments to ensure comparability across schools and time points. Sustained support for longitudinal monitoring is necessary to detect emerging patterns and to evaluate intervention effectiveness. At the research level, results emphasize the need of multi-method designs in capturing the complexity of bullying and cyberbullying, particularly given the potential for measurement artifacts to obscure true developmental trajectories.

## Supplementary Information

Supplementary Material 1.

## Data Availability

The datasets used and analysed during the current study are available from the corresponding author on reasonable request.
