# Supplementary material for: Longitudinal pattern of bullying and cyberbullying victimization and perpetration in adolescence: a multi-method approach from the Bullying and Youth Mental Health Naples Study (BYMHNS)
Source: BMC Psychol. 2026 Jul 3;14:1380. doi: 10.1186/s40359-026-04885-9 (PMC13602577; doi:10.1186/s40359-026-04885-9)
Supplement: Supplementary file 1 [file 40359_2026_4885_MOESM1_ESM.docx]

**Suppplementary Materials**

***Correlation between single-item and multi-item bullying and cyberbullying (victimization and perpetration) assessment at both Time 1 and Time 2***

Table S1 displayed the correlations between single-item and multi-item measures of bullying for victimization and perpetration. At both T1 and T2, data showed that single-item victimization index was strongly correlated with the multi-item victimization score (T1: r = .506, N = 788, p<.001; T2: r = .484, N = 776, p<.001) than multi-item perpetration score (T1: r = .179, N = 788, p<.001; T2: r = .082, N = 776, p<.05) and that single-item perpetration index was strongly correlated with the multi-item perpetration score (T1: r = .452, N = 788, p<.001; T2: r = .363, N = 779, p<.001) than multi-item victimization score (T1: r = .286, N = 788, p<.001; T2: r = .246, N = 779, p<.001).

**Table S1.** Correlation between single-item and multi-item bullying assessment at both Time 1 and Time 2

| Time 1 | | |
| --- | --- | --- |
| Scale | Bullying | |
|  | Victimization  (N = 788) | Perpetration  (N = 788) |
| MPVS |  |  |
| Physical Victimization | .302*** | .214*** |
| Social Manipulation | .387*** | .246*** |
| Verbal Victimization | .479*** | .195*** |
| Attacks on Property | .287*** | .186*** |
| Total score | .506*** | .286*** |
|  |  |  |
| IBS-B |  |  |
| Total score | .179*** | .452*** |
|  |  |  |
| Time 2 | | |
| Scale | Bullying |  |
|  | Victimization  (N = 776) | Perpetration  (N = 779) |
| MPVS |  |  |
| Physical Victimization | .308*** | .243*** |
| Social Manipulation | .308*** | .131*** |
| Verbal Victimization | .499*** | .221*** |
| Attacks on Property | .259*** | .132*** |
| Total score | .484*** | .246*** |
|  |  |  |
| IBS-B |  |  |
| Total score | .082* | .363*** |

Note 1. MPVS = Multidimensional Peer Victimization Scale; IBS-B = Bully subscale of Illinois Bully Scale.

Note 2. ***FDR corrected p<.001 within each table; *FDR corrected p<.05 within each table.

Table S2 displayed the correlations between single-item and multi-item measures of cyberbullying for victimization and perpetration. At both T1 and T2, data showed that single-item victimization index was strongly correlated with the multi-item victimization score (T1: r = .469, N = 788, p<.001; T2: r = .428, N = 779, p<.001) than multi-item perpetration score (T1: r = .241, N = 788, p<.001; T2: r = .105, N = 780, p<.01) and that single-item perpetration index was strongly correlated with the multi-item perpetration score (T1: r = .465, N = 788, p<.001; T2: r = .354, N = 770, p<.001) than multi-item victimization score (T1: r = .313, N = 788, p<.001; T2: r = .267, N = 771, p<.001).

**Table S2.** Correlation between single-item and multi-item cyberbullying assessment at both Time 1 and Time 2

| Time 1 |  | |
| --- | --- | --- |
| Scale | Cyberbullying | |
|  | Victimization  (N = 788) | Perpetration  (N = 788) |
| Cybervictimization | .469*** | .313*** |
| Cyberbullying | .241*** | .465*** |
|  |  |  |
| Time 2 |  | |
| Scale | Cyberbullying | |
|  | Victimization | Perpetration |
| Cybervictimization | .428***^a^ | .267***^c^ |
| Cyberbullying | .105**^b^ | .354***^d^ |

Note 1. ***FDR corrected p<.001 within each table; **FDR corrected p<.01 within each table.

Note 2. ^a^N = 779; ^b^N = 780; ^c^N = 771; ^d^N = 770.

**Table S3.** Correlation between single-item bullying and cyberbullying (victimization and perpetration) at both Time 1 (N = 788) and Time 2

| Time 1 |  |  |  |  |
| --- | --- | --- | --- | --- |
|  | Bullying Victimization | Bullying Perpetration | Cyberbullying Victimization | Cyberbullying Perpetration |
| Bullying Victimization | - |  |  |  |
| Bullying Perpetration | .277*** | - |  |  |
| Cyberbullying Victimization | .283*** | .122*** | - |  |
| Cyberbullying Perpetration | .173*** | .316*** | .257*** | - |
| Time 2 |  |  |  |  |
|  | Bullying Victimization | Bullying Perpetration | Cyberbullying Victimization | Cyberbullying Perpetration |
| Bullying Victimization | - |  |  |  |
| Bullying Perpetration | .275***^a^ | - |  |  |
| Cyberbullying Victimization | .298***^b^ | .031^d^ | - |  |
| Cyberbullying Perpetration | .124**^c^ | .189***^e^ | .226***^f^ | - |

Note 1. ***FDR corrected p<.001 within each table; **FDR corrected p<.01 within each table.

Note 2. ^a^N = 772; ^b^N = 771; ^c^N = 761; ^d^N = 774; ^e^N = 764; ^f^N = 766.

**Table S4.** Correlation between multi-item bullying and cyberbullying (victimization and perpetration) at both Time 1 (N = 788) and Time 2

| Time 1 |  |  |  |  |
| --- | --- | --- | --- | --- |
|  | MPVS | IBS-B | Cybervictimization | Cyberbullying |
| MPVS | - |  |  |  |
| IBS-B | .356*** | - |  |  |
| Cybervictimization | .452*** | .419*** | - |  |
| Cyberbullying | .187*** | .634*** | .509*** | - |
| Time 2 |  |  |  |  |
|  | MPVS | IBS-B | Cybervictimization | Cyberbullying |
| MPVS | - |  |  |  |
| IBS-B | .333***^a^ | - |  |  |
| Cybervictimization | .493***^b^ | .431***^b^ | - |  |
| Cyberbullying | .228***^c^ | .595***^c^ | .453***^d^ | - |

Note 1. MPVS = Multidimensional Peer Victimization Scale; IBS-B = Bully subscale of Illinois Bully Scale.

Note 2. ***FDR corrected p<.001 within each table.

Note 3. ^a^N = 787; ^b^N = 784; ^c^N = 785; ^d^N = 783.
